# Burnout Integrative Measure: A preliminary validation among French college students

**DOI:** 10.3389/fpsyg.2022.904367

**Published:** 2022-11-09

**Authors:** Sophie Berjot, Tiphanie Weber, Tiphaine Huyghebaert-Zouaghi

**Affiliations:** Laboratoire C2S (Cognition Santé Société) (EA 6291), Université de Reims Champagne Ardenne, Reims, France

**Keywords:** burnout, students, college, detachment, weariness, inadequacy

## Abstract

The aim of this research was to create and validate an integrative measure of college students’ burnout. A burnout measure was proposed and extended the three-dimensional conceptualization of burnout (weariness, detachment toward social objects, inadequacy). Based on prior research, our conceptualization and measure distinguished between types of weariness (cognitive, physical, and emotional weariness) and between different targets of detachment toward social objects (studies, other students, teachers). We also relied on negatively worded items to assess inadequacy, as suggested in the literature. The criterion-related validity of our Burnout Integrative Measure (BIM) was examined by exploring associations with two closely related constructs, namely stress and depression. The participants are 905 students in several disciplinary (psychology, nursing care, medicine, science and techniques in sports and physical activities). Results from structural equation modelling provided support for a third-order model encompassing the different targets of detachment, the distinct types of weariness, and inadequacy. The third-order model had a better fit than a first-order model (with a global burnout) and a second-order model (with no distinction between the targets of detachment and the types of weariness). Correlations with related constructs (depression and stress) mostly confirmed our hypotheses. Results and practical implications are discussed.

## Introduction

Burnout was initially described as “a syndrome of emotional exhaustion and cynicism that occurs frequently among individuals who ‘do people-work’ of some kind” (Maslach and Jackson, 1981, p. 99). Maslach and Jackson (1981) identified three dimensions of human service workers’ burnout: *emotional exhaustion*, *depersonalization* and *reduced personal accomplishment*. The Maslach Burnout Inventory-Human Services Survey (MBI-HSS; Maslach and Jackson, 1996) was thus developed and validated to measure these dimensions in human service workers. Later, burnout was revealed not to be specific to human service workers and was refined to be applied to the other workers. Because of this change in scope, symptoms’ definitions evolved, as well as their assessment, through a new version of the Maslach Burnout Inventory (MBI). This scale, the MBI-General Survey (MBI-GS, Schaufeli et al., 1996), assesses the three dimensions of workers’ burnout: *exhaustion*, *cynicism* and *a lack of professional efficacy*. *Exhaustion* is not only defined by emotional exhaustion as it was the case for human services workers but takes also into account physical exhaustion (Maslach et al., 2001). *Cynicism* represents a cold and detached attitude toward work as a whole, not toward recipients as it was the case for the corresponding depersonalization dimension of the MBI-HSS. *A lack of professional efficacy* is defined by feelings of being less effective in one’s work, not in one’s work with recipients as it was the case for the corresponding personal accomplishment dimension of the MBI-HSS. Note however that this three dimensional conceptualization of burnout, even if largely widespread, is not the only one conceptualization of burnout among workers as it coexist with other conceptualization such as the bidimensional conceptualization of Halbesleben and Demerouti (2005) or more recently the four dimensional conceptualization of Schaufeli et al. (2020).

Although burnout has been described and defined within the professional context, several studies have suggested that it could develop in other contexts which have similarities. Burnout has been studied in the parental context with the maternal burnout syndrome (Lebert-Charron et al., 2018) and in the academic context (Schaufeli et al., 2002; Salmela-Aro et al., 2008; Faye-Dumanget et al., 2017). It means that students can also develop burnout. Indeed, just like workers, students are embedded within a social system with classmates instead of colleagues, teachers instead of immediate supervisors, administration (specifically the head of the institution) instead of senior management. Logically, a few tools have been developed to assess burnout within the academic context, including some in the French language, but these tools suffer from some drawbacks, making their use problematic. Therefore, the present research aimed to address these disadvantages by creating and validating a new tool (i.e., the Burnout Integrative Measure, BIM) to assess burnout among French college students. Precisely, this research intended to examine the construct validity of the BIM and to provide preliminary evidence of its criterion-related validity.

### Existing measures of student burnout

The Maslach Burnout Inventory-Student Survey (MBI-SS, Schaufeli et al., 2002) is a direct translation of the MBI-GS to the academic context, with the items referring to work being adapted to refer to studies. Therefore, this scale assesses burnout with the same underlying conceptualization as the one proposed for workers (i.e., exhaustion, cynicism towards studies and reduced efficacy). The MBI-SS has been translated and validated in French (Faye-Dumanget et al., 2017) among students aged from 18 to 25 years old, thus allowing its use in the academic context. Yet, several MBI tools are copyrighted and thus not openly or easily accessible to researchers and practitioners.

An alternative is the Genoud and Reicherts’ Burnout Scale for students (2008), which is an adaption of several MBI versions to the academic context. It also goes further by proposing to distinguish teachers from classmates by considering them as distinct targets of depersonalization. Unfortunately, despite this interesting and promising theoretical development, their study did not allow the authors to validate the scale. Authors explained failing to reach satisfactory results in terms of factorial structure and scale homogeneity (Genoud and Reicherts, 2008).

To our knowledge, the only other existing tool available in French to assess student burnout is the School Burnout Inventory (Salmela-Aro et al., 2009), which was translated and validated in French by Meylan et al. (2015). According to Salmela-Aro et al. (2008, p.664), «school burnout is defined along three dimensions: exhaustion due to school demands; cynical and detached attitude towards one’s school; and feelings of inadequacy as student». In developing this scale, authors addressed a shortcoming of the MBI-SS, which measures reduced professional efficacy through positively worded items which actually tap into a positive psychological experience. Yet reverse coding positive items does not equal measuring a negative psychological experience such as a burnout symptom. Therefore, in the School Burnout Inventory, the inadequacy dimension (corresponding to the reduced professional efficacy dimension in the MBI-SS) is assessed *via* negatively worded items, thus better reflecting this adverse experience. Unfortunately, the French version was validated among students aged between 13 and 17 years old. As such, this tool has been validated for teenagers and is thus not suitable for older students. It should be noted that a version for older students was developed and validated (i.e., Study Burnout Inventory, Salmela-Aro and Read, 2017). However, to the best of our knowledge, this scale has not been validated in French.

In sum, although the above reviewed tools present some advantages, they also have some practical, methodological, and theoretical drawbacks. One of them presents a practical limitation, because of restrictions of use (i.e., MBI-SS). The other two have methodological issues either because they could not be psychometrically validated (i.e., the Genoud and Reicherts’ Burnout Scale) or because of the population that was used for validation (i.e., the School Burnout Inventory). These tools also raise some theoretical questions in their consideration of the dimensions of burnout, which we further develop in the following section.

### Beyond existing measures of student burnout: Multidimensionality of the BIM

First, the above-mentioned tools all assess students’ general exhaustion (Genoud and Reicherts, 2008; Meylan et al., 2015; Faye-Dumanget et al., 2017). However, other conceptualizations suggest that there are distinctive types of exhaustion as emphasized in Pines and Kafry’s (1981) work or, more recently, by Halbesleben and Demerouti (2005). These authors, in line with the MBI framework, confirmed the importance of emotional and physical fatigue, but also mentioned the importance of cognitive or mental fatigue. This distinction between physical, emotional, and mental exhaustion is also present in Shirom and Melamed’s (2006) conceptualization of burnout. Yet, to the best of our knowledge, this distinction between different types of exhaustion (i.e., emotional, physical, cognitive) has never been applied to burnout within the educational setting. Nonetheless, the consideration of physical and cognitive weariness, in addition to the most commonly examined emotional exhaustion dimension (i.e., not be able to feeling something), appears of particular relevance when considering students’ burnout. Indeed, the academic context is a demanding one: students have to attend classes but also to complete assignments or study for exams during evenings and weekends (Salmela-Aro and Read, 2017). This intensive schedule leaves them with few opportunities for recovery and may thus drain their physical energy to the point where it cannot be restored. As such, physical weariness implies an intense tiredness which does not disappear even with enough sleep and recovery. Moreover, students have to maintain a high level of attention and concentration during classes and homework, and evolve in an evaluative context, which may drain their cognitive energy. Cognitive weariness can thus manifest itself through difficulties of attention and concentration on daily academic tasks. It therefore seems important to distinguish the three above-mentioned types of fatigue which we propose to all label weariness, in order to distinguish from normal fatigue and refer to a more intense and adverse drain of energy, be it cognitive, physical or emotional.

Second, regarding the cynicism/depersonalization dimension, as previously mentioned, tools often only assess one target at a time and this target most often is one’s studies. The Genoud and Reicherts’ scale (2008) was, in this respect, quite progressive as it assessed different targets of depersonalization (i.e., studies, teachers and classmates). However, considering that the depersonalization/cynicism dimension is a negative and detached attitude (Maslach et al., 2001; Schaufeli et al., 2002), it can thus theoretically express itself toward several types of social objects that are present in one’s environment, and that are pertinent as they represent a threat to students’ well-being or identity. This distinction between social objects that one can detach themselves from is consistent with prior research (e.g., Golembiewski et al., 1983; Demerouti et al., 2005; Genoud and Reicherts, 2008) arguing that these distinct targets must be considered to give a more comprehensive overview of cynicism. Given this more general definition, and to consider different types of social objects, in our research this dimension will be called *detachment toward social objects.* Precisely, we conceptualize detachment as the endpoint of one’s chronic use of disengagement strategies from one or several targets that are present in their environment, in reaction to threat, especially identity threat (Crocker and Major, 1989; Steele and Aronson, 1995; Lesage et al., 2013). The idea is that the sources of threat would also be the targets of disengagement and thus become the targets of detachment.

Third, the lack of personal accomplishment/reduced self-efficacy dimension of burnout is usually assessed using positively worded items. This can be problematic as suggested by several authors (Bouman et al., 2002) who insist that it is important to distinguish lack of efficacy from inefficacy. Indeed, Bresó et al. (2007, p.472) note that “the relatively strong correlations of the inefficacy scale with both remaining burnout dimensions support the conceptualization of academic burnout as a three-dimensional syndrome constituted by exhaustion, cynicism, and academic inefficacy, instead of (reversed) efficacy.” In other words, low or reversed efficacy is not equivalent to the detrimental experience of inefficacy. Indeed, these constructs reflect two distinct psychological experiences: one refers to a lack of positive experience (i.e., reduced self-efficacy), while the other reflects an adverse psychological experience (i.e., inefficacy). To avoid this confusion and to tap more directly into this negative psychological experience, the use of negative statement was chosen in the present research, as it was done in the works of Salmela-Aro et al. (2009). Moreover, to express more fully the content of the items and the negative psychological experience reflected in this dimension, this third dimension was relabelled *inadequacy* in the present research, in line with Salmela-Aro and colleagues (Salmela-Aro et al., 2008; Salmela-Aro and Read, 2017).

In sum, based on previous criticisms addressed to the MBI scales and building upon other theoretical models, modified definitions of the three dimensions of student burnout were proposed and were consequently renamed *weariness*, *detachment toward social objects* and *inadequacy* in order to better reflect their proposed conceptualization. We offered to develop the Burnout Integrative Measure (BIM) to operationalize these new definitions.

### Uncovering the BIM’s indirect criterion-related validity

The aim of this study was to test the structure of the BIM and to highlight correlations between each subscale. Previous studies highlighted a higher correlation between exhaustion and cynicism than between exhaustion and academic efficacy (Schaufeli et al., 2002; Faye-Dumanget et al., 2017) which is why high correlations between each type of weariness and each target of detachment were expected. Based on Bresó et al. (2007) and Maroco et al. (2014) who found higher correlations between (emotional) exhaustion, cynicism and (academic) inefficacy than between (emotional) exhaustion, cynicism and (academic) efficacy, strong links between each subscale were expected.

In the present research, the scale’ indirect criterion-related validity was tested using depression and perceived stress as correlates because of their importance in the academic domain (Grebot and Barumandzadeh, 2005; Beiter et al., 2015). Moreover, these variables are known to strongly relate to burnout (Schaufeli and Enzmann, 1998). Indeed, depression is recognized to be closely and positively related to burnout, in particular to the exhaustion dimension (Bianchi et al., 2015). As such, a strong relation between depression and weariness and moderate to low relations with the two other dimensions were expected. Stress is also strongly related to burnout, considered by some authors as the principal antecedent of burnout (Maslach et al., 2001). Therefore, based on previous work, strong relations between stress and different facets of weariness were expected (Lesage et al., 2013), and moderate relations between stress and the two other dimensions.

## Materials and methods

### Participants and procedure

Paper and online questionnaire surveys were collected by the first author and two research assistants from 905 French college students, including 587 women (64.86%) and 289 males (31.93%). Twenty-nine participants (3.20%) did not wish to indicate their gender. Students were either in their first year of psychology (*N* = 256), of Science and Techniques in Sports and Physical Activities (STAPS, *N* = 255), in their second year of nursing school (*N* = 72), and in medicine either in their first-year (*N* = 191) or later (2^nd^ to 6^th^ year, *N* = 130). One participant did not indicate their major. Respondents’ mean age was 19.15 (*SD_Age_* = 1.74), the youngest being 17 and the oldest 26. A total of 36 participants (3.98%) did not wish to indicate their age. All were recruited online or during lectures and were assured of the voluntary and anonymous nature of their participation. Out of the 905 surveyed college students who all completed the burnout measure, 328 also completed the perceived stress scale and 256 also completed the depression scale^1^. Our study was presented as research on students’ feelings about their ongoing studies. This study was considered not to need approval from the institution ethics committee according to local regulations.

### Measures

**Burnout** was measured through the 28-item final version of the BIM (α = 0.95). Based on the literature, a pool of 29 items was created. Twelve items assessed the dimension of detachment toward social objects, 4 for detachment toward other students (OS1 to 4), 5 for detachment toward studies (STU1 to 5) and 4 for detachment toward teachers (TEA1 to 4). Twelve items assessed weariness, 4 for each type of weariness: cognitive (COG1 to 4), physical (PHYS1 to 4) and emotional (EMO1 to 4). Four items assessed inadequacy (INA1 to 4). Their face-validity was tested and then, a principal component analysis was run to test its structure in a prior study with first year students (N = 297). The results of this preliminary study showed a satisfactory structure of the scale. The factorial analysis (principal component with oblimin rotation) showed 5 distinct factors that explained 57.52% of the total variance. All items loaded on their respective dimension except for EMO3 which loaded on the inadequacy dimension, CAM1 which had a low loading and ETU1 which had a low loading on another dimension). So, ETU1 was deleted for the following study (CAM1 and EMO3 were kept because their formulation was judged adequate). Alphas were all satisfactory, going from.83 to.94. Participants had to indicate their degree of agreement with each statement using a six-point Likert scale ranging from 0 (“Do not agree at all”) to 5 (“Totally agree”).

**Depression** was assessed with The Beck Depression Inventory-Fast Screen-France (BDI-FS-Fr), validated by Alsaleh and Lebreuilly (2017). This scale is composed of seven items (α = 0.78) and does not take the somatic complaints into account. Participants answered using a four-point Likert-type scale.

**Perceived stress** was assessed with The Perceived Stress Scale-10 (α = 0.83) validated in French by Lesage et al. (2012). Participants had to indicate how frequently they experienced each statement during the last two weeks using a five points Likert-type scale ranging from 0 (“Never”) to 4 (“Often”).

## Results

Confirmatory factor analyses were run using AMOS version 24 to test the structure of the BIM. Several indices were used to assess model fit such as the chi-square (χ^2^), the degree of freedom (*df*), the Comparative Fit Index (CFI), the Tucker Lewis Index (TLI), the Root Mean Square Error of Approximation (RMSEA) and the Akaike Information Criterion (AIC).

**Figure 1:**
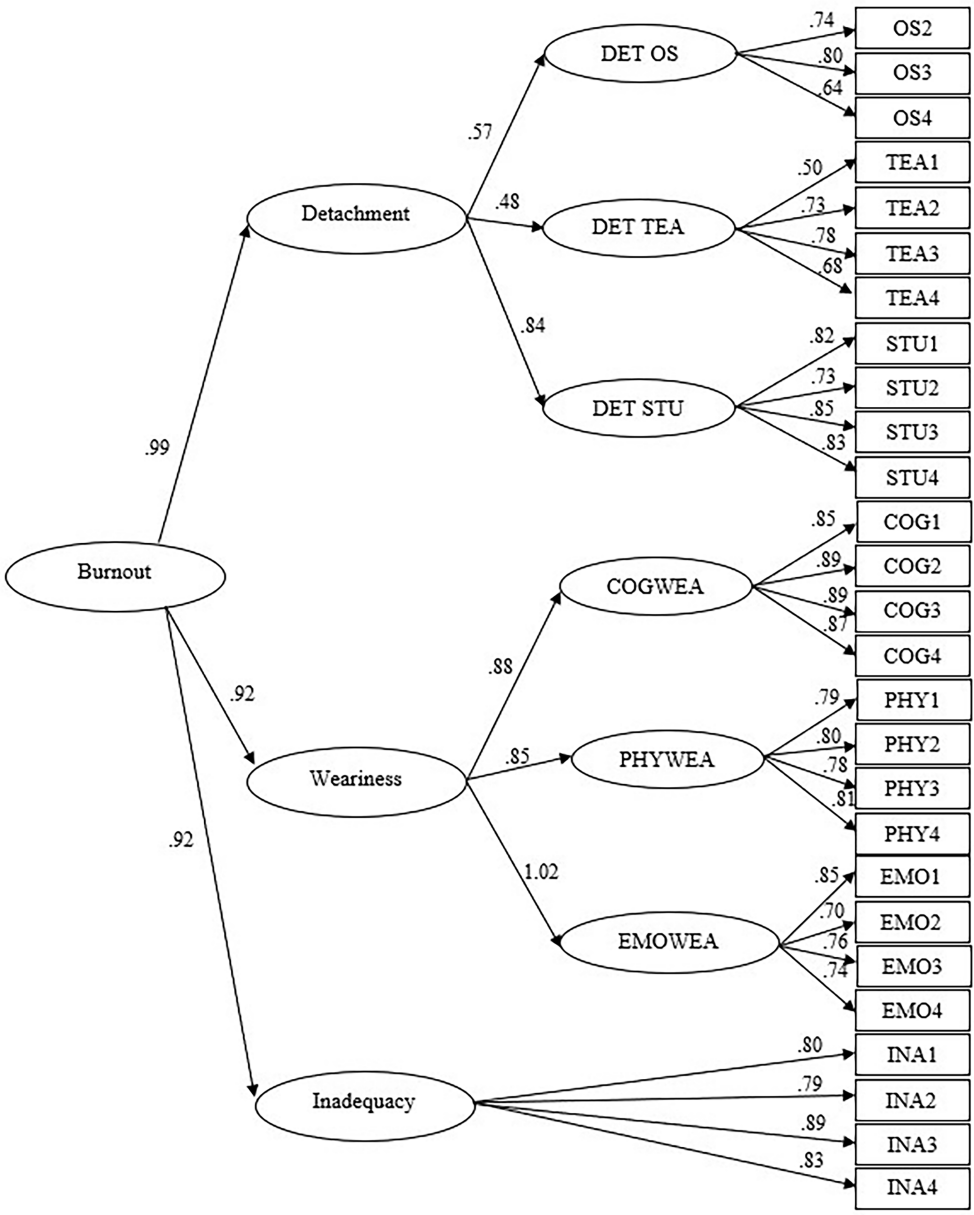
Burnout Model 4 (BM4). OS = Other students; TEA = Teachers; STU = Studies; COG = Cognitive; PHY = Physical; EMO = Emotional; INA = Inadequacy; DET OS = Detachment toward other students; DET TEA = Detachment toward teachers; DET STU = Detachment toward studies; COG WEA = Cognitive weariness; PHY WEA = Physical weariness; EMO WEA = Emotional weariness. All links are significant. OS1 was removed after the analysis of BM1.

Four models were tested, covariances between errors were not allowed in these models^2^. The first model had all items loading on a burnout latent variable (Burnout Model 1, BM1). One of the items designed to assess detachment toward other students had a low factor loading, therefore this item was removed and a second model was tested. This item was removed in the three other models. The second model was identical to BM1 except that it did not include the problematic item identified in BM1 (Burnout Model 2, BM2). The third model had each item load on its corresponding dimension (detachment, weariness, and inadequacy) as a latent variable, and each dimension load on burnout as second order variable (Burnout Model 3, BM3). The fourth model had each item loading on its corresponding dimension as a latent variable (detachment toward other students, detachment toward teachers, detachment toward studies, cognitive weariness, physical weariness, emotional weariness, and inadequacy), each of the three detachment and the three weariness dimensions loaded on their respective detachment and weariness second order variable. The detachment, weariness, and inadequacy latent variables loaded on a burnout third order variable (Burnout Model 4, BM4). Results from all models are displayed in Table 1 and indicate that BM4 presented the best fit to the data. This model (BM4) was thus retained for subsequent analyses (Figure 1).

**Table 1:** Results from confirmatory factor analyses.

	χ^2^	*df*	TLI	CFI	RMSEA	AIC	Comparison model	Δ χ^2^	Δ*df*
BM1	5061.27	350	0.70	0.72	0.12	5173.27			
BM2	4951.18	324	0.71	0.73	0.13	5059.18	BM1 vs. BM2	110.09**	26
BM3	3544.48	321	0.79	0.81	0.11	3658.48	BM2 vs. BM3	1406.70**	3
BM4	1853.24	315	0.90	0.91	0.07	1979.24	BM3 vs. BM4	1691.24**	6

CFI = Comparative Fit Index; TLI = Tucker-Lewis fit Index; RMSEA = Root Mean Square Error of Approximation; AIC = Akaike Information Criterion; Δ χ^2^ = Chi-square difference; Δdf = degree of freedom difference; BM1 = Burnout Model 1; BM2 = Burnout Model 2; BM3 = Burnout Model 3; BM4 = Burnout Model 4. ** p < 0.01.

Albeit the low number of participants in each group, an invariance analysis was run according to gender. The difference of χ^2^ (571.4; df = 315) was significant at *p* < 0.001. However, all indices were still better than those of other models (respectively for women and men, TLI = 0.893 and 0.869; CFI = 0.904 and 0.88; RMSEA = 0.078 and.077; AIC = 1551.75 and 977.42). Only the item ENS1 had a lower coefficient among men (0.42).

Correlations between sub-dimensions are presented in Table 2.

**Table 2:** Correlations between variables.

Variable	*M*	*SD*	1	2	3	4	5	6	7	8	9
1. Detachment toward OS	1.40	1.35	–								
2. Detachment toward TEA	0.92	0.93	0.24	–							
3. Detachment toward STU	0.77	1.11	0.43	0.43	–						
4. Cognitive weariness	1.94	1.48	0.40	0.46	0.56	–					
5. Physical weariness	2.11	1.54	0.42	0.45	0.48	0.73	–				
6. Emotional weariness	1.32	1.29	0.49	0.46	0.68	0.79	0.76	–			
7. Inadequacy	1.39	1.39	0.47	0.39	0.69	0.69	0.60	0.75	–		
8. Depression	0.46	0.46	0.41	0.26	0.44	0.53	0.56	0.48	0.55	–	
9. Stress	1.77	0.73	0.34	0.24	0.34	0.46	0.51	0.40	0.48	0.60	–

OS = Other students; TEA = Teachers; STU = Studies. Variables 1 to 7 ranging from 0 to 5, depression ranging from 0 to 3 and stress from 0 to 4.

The three types of weariness were significantly related to depression (see Table 2). Physical weariness presented the strongest association with depression (*r* = 0.56), followed by inadequacy (*r* = 0.55), cognitive weariness (*r* = 0.53), emotional weariness (*r* = 0.48), and by the distinct types of detachment toward social objects (from 0.26 to 0.44).

The pattern of results for stress thus appeared to be almost identical to that of depression (see Table 2). Results showed stress to be moderately to strongly associated with each weariness dimension (ranging from 0.40 to 0.51), with detachment toward social objects (ranging from 0.24 to 0.34), and with inadequacy (*r* = 0.48).

**Figure 2:**
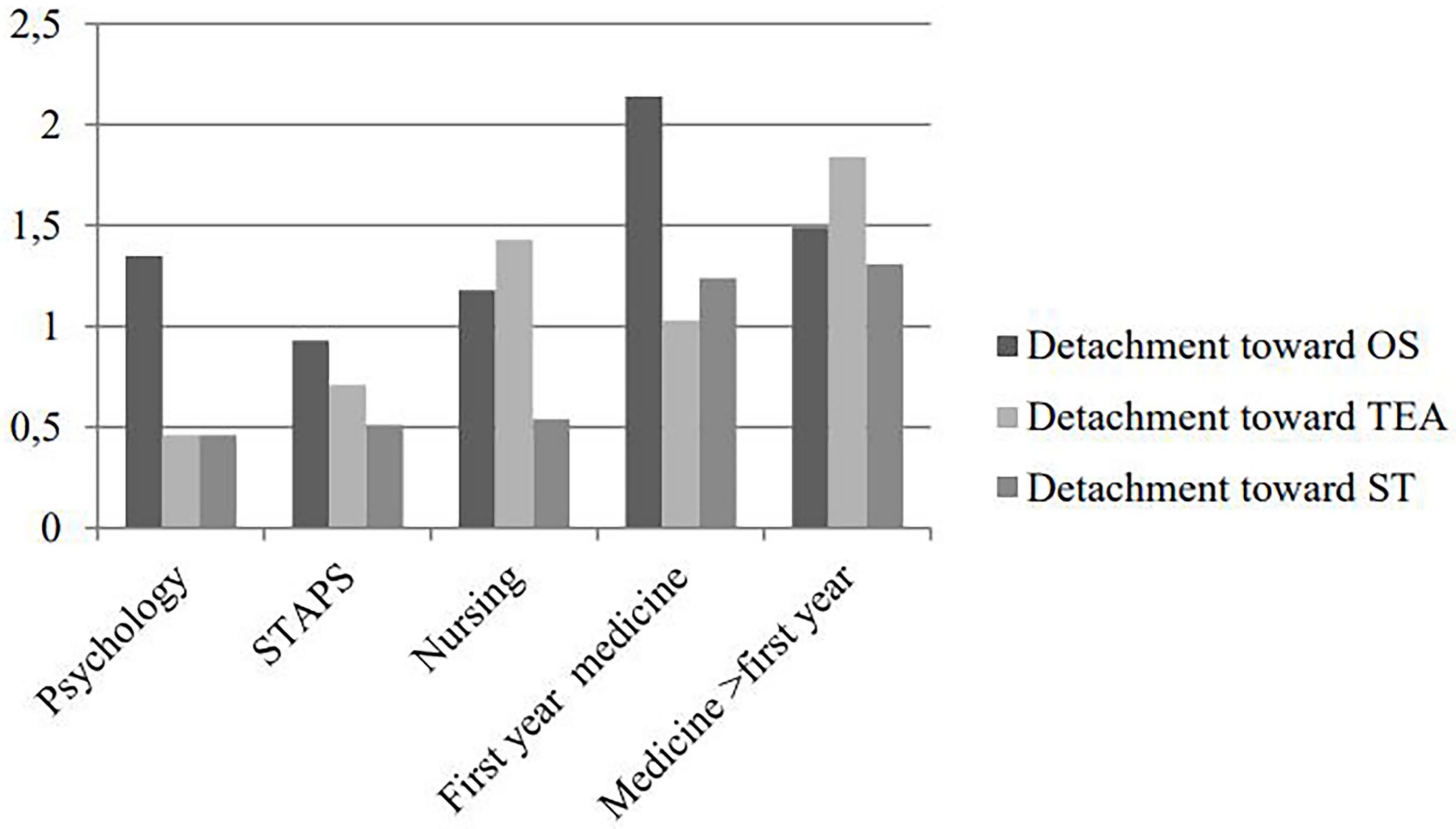
Means of detachment toward other students, toward teachers and toward studies for each disciplinary.

Complementary analyses were conducted to compare levels of burnout (mean of each symptom) and symptoms of burnout of students according to their discipline and their gender. Anovas highlighted significant differences between students for burnout and for each symptom (see Table 3). There were also significant differences for detachment toward other students, toward teachers, toward studies, cognitive weariness, physical weariness and emotional weariness (see Figure 2).

**Table 3:** Comparison of burnout levels and burnout symptoms levels of students who come from several disciplinary (LSD test).

Variable	*M*(*SD*) psychology	*M*(*SD*) STAPS	*M*(*SD*) nursing	*M*(*SD*) medicine (first year)	*M*(*SD*) medicine (>first year)	*F*(4,899)
Detachment toward OS	1.35a (1.28)	0.93b (1.14)	1.18a, b (1.29)	2.14c (1.41)	1.49a (1.34)	25.38**
Detachment toward TEA	0.46a (0.55)	0.71b (0.72)	1.43c (0.97)	1.03d (0.86)	1.84e (1.12)	76.31**
Detachment toward STU	0.46a (0.86)	0.51a (0.84)	0.54a (0.86)	1.24b (1.44)	1.31b (1.16)	28.62**
Detachment	0.76a (0.67)	0.72a (0.67)	1.05b (0.63)	1.47c (0.99)	1.55c (0.92)	46.94**
Cognitive weariness	1.13a (1.09)	1.38b (1.15)	1.90c (1.26)	3.31d (1.24)	2.67e (1.43)	116.09**
Physical weariness	1.43a (1.19)	1.21b (1.07)	2.43c (1.38)	3.35d (1.32)	3.22d (1.36)	130.19**
Emotional weariness	0.66a (0.85)	0.76a (0.90)	1.21b (1.04)	2.54c (1.31)	2.00d (1.21)	122.58**
Weariness	1.08a (0.91)	1.12a (0.89)	1.85b (1.06)	3.07c (1.13)	2.63d (1.19)	158.76**
Inadequacy	0.96a (1.06)	0.86a (1.03)	1.14a (1.25)	2.46b (1.55)	1.84c (1.40)	60.56**
Burnout	0.93a (0.78)	0.90a (0.75)	1.34b (0.80)	2.33c (1.08)	2.01d (1.03)	107.03**

OS = Other students; TEA = Teachers; STU = Studies. For each line, the use of the same letter indicates that there is not a significant difference between the groups. ^**^p < 0.01.

There were also significant differences between gender for all sub-dimensions except for detachment toward teachers (see Table 4). Means were higher for the female’ students than for male’ students.

**Table 4:** Comparison of burnout (sub-)dimensions means between genders.

Variable	*M*(*SD*) female	*M*(*SD*) male	*t*(874)
Detachment toward OS	1.53 (1.37)	1.82 (1.29)	3.59**
Detachment toward TEA	0.92 (0.96)	0.97 (0.87)	−0.70
Detachment toward STU	0.83 (1.17)	0.64 (0.96)	2.37*
Detachment	1.09 (0.89)	0.93 (0.79)	2.64**
Cognitive weariness	2.15 (1.54)	1.58 (1.29)	5.36**
Physical weariness	2.45 (1.57)	1.47 (1.28)	9.21**
Emotional weariness	1.49 (1.38)	1.02 (1.06)	5.13**
Weariness	2.03 (1.38)	1.35 (1.07)	7.27**
Inadequacy	1.58 (1.48)	1.01 (1.13)	5.81**
Burnout	1.57 (1.13)	1.10 (0.89)	6.20**

OS = Other students; TEA = Teachers; STU = Studies. ^**^p < 0.01; ^*^p < 0.05.

## General discussion

The aim of this research was to create and validate an Integrative Measure of Burnout for college students in the academic context. This measure was developed to assess detachment toward social objects (depersonalization/cynicism), weariness (exhaustion) and inadequacy (reduced professional efficacy), and was underpinned by extended conceptualizations for each dimension.

### Theoretical implications

Results from confirmatory factor analyses confirmed our conceptual modelling of detachment toward social objects and weariness, by showing the superiority of a third-order model with weariness, detachment toward social objects and inadequacy as latent variables. Our results therefore confirm the three-component model of burnout. They also highlight the importance to distinguish between distinct forms of detachment (i.e., detachment toward other students, teachers and studies) and between different manifestations of weariness (i.e., cognitive, physical and emotional weariness) which confirms the propositions of Genoud and Reicherts (2008), Demerouti et al. (2005) and Shirom and Melamed (2006).

Correlations between subscales were a little surprising as shown by the low to moderate links between different types of detachment toward social objects. It seems that a student can be detached from teachers but not necessarily from other students. However, they were in line of those found by Demerouti et al. (2005) in the professional context.

Results showed that, while detachment toward other students and toward teachers were moderately linked with all other dimensions of burnout, detachment toward studies was strongly linked to most of them (i.e., cognitive and emotional weariness, and inadequacy). This means that when a student is detached from his studies, he could also present difficulties to concentrate on tasks, feel emotional drain and think that he is incompetent. Inadequacy was strongly correlated to cognitive and emotional weariness while being moderately correlated with the most of the other dimensions of burnout. These results are similar to those of Faye-Dumanget et al. (2017) who found academic efficacy to be strongly linked with cynicism (toward studies). However, our results showing moderate to high correlations for weariness differed from those of Faye-Dumanget et al. (2017) who found weak correlation between academic efficacy and exhaustion. One of the reasons may lay in the negative wording of the inadequacy scale compared to the positive wording of academic efficacy scales. Indeed, Bresó et al. (2007)‘s study also presented stronger correlations between exhaustion and cynicism when they used an inefficacy scale rather than an efficacy measure.

Correlations with other constructs were in the expected direction except for inadequacy which showed higher correlations than expected with stress and depression. More precisely, the more students are stressed, the more they feel cognitive and physical weariness as well as inadequacy. The more they feel depressed, the more they feel cognitive, emotional, and physical weariness as well as inadequacy. Once again, one explanation of our results could lay in our studying inefficacy (i.e., an adverse experience) rather than reduced efficacy (i.e., lack of a positive experience). Future studies should thus further investigate the relationship between inadequacy, depression and stress among students to confirm their strong links.

Finally, the difference between students’ levels on inadequacy is in line with the results found by Faye-Dumanget et al. (2018) except for the first-year students in medicine. Regarding the other dimensions, they did not find differences for exhaustion and cynicism, while in the present research, we did find such differences. Maybe it could be explained by the use of different tools or the population under study (university and prep school for Faye-Dumanget et al., and almost only university students for this study). Our results also highlight the pertinence of distinguishing targets of detachment. Indeed here, students from different majors did not detach from the same targets. While advanced students in medicine detached themselves from teachers, this was not the case of first year students who instead presented a detachment from other students. This may be explained by the very high numbers of students in the first year of medicine coupled with the competition climate that is often the rule within this discipline. So, these results show the importance of distinguishing between targets of detachment that seem to reflect the specific reactions to their personal experience of studies within their domain.

In sum, our results showed satisfactory psychometric properties of the Burnout Integrative Measure. This new measure is composed of three subscales (i.e., weariness, detachment toward social objects, and inadequacy) representing the three symptoms of burnout. Our measure extends upon prior conceptualizations by 1) distinguishing between targets of detachment as suggested by some authors (e.g., Genoud and Reicherts, 2008); 2) differentiating between three types of weariness (i.e., emotional, cognitive, and physical) and, by doing so, integrating crucial information from the Shirom-Melamed Burnout Measure (Shirom and Melamed, 2006) and making it complementary with the MBI approach, and 3) negatively wording the inadequacy items, in order to better tap into the adverse experience that it represents, whereas prior work considered this dimension as lack of a positive experience (Schaufeli et al., 2002). As such, our work extends upon prior research on students’ burnout and contributes to bridge the gap between opposite, yet popular, approaches of burnout (Maslach and Jackson, 1981; Maslach et al., 1996; Shirom and Melamed, 2006).

### Limitations and research perspectives

Despite its contribution to the literature on student burnout, this research presents some limitations. First, the validity of the BIM for College Students was not compared to other scales assessing student burnout in French. Yet, it should be noted that this choice was motivated by the limitation of the use of MBI (i.e., copyrighted) and by the population in which the School Burnout Inventory was validated (i.e., young adolescents). Depression was used to test indirect criterion validity in reference to the debate in the literature about the proximity of the two concepts (Bianchi et al., 2015; Parker and Tavella, 2021). It is important to note however that many studies showed that depression, at least among employees, is more a consequence of burnout than a component (because of its links with exhaustion/weariness). It is then important to explore more fully, using a longitudinal design, how burnout, measured with the BIM, can predict depression and/or it is a component of burnout.

Second, data were collected at the beginning of the first semester for students in psychology, STAPS and nursing and at the second semester for medicine students. Maricuțoiu and Sulea’s study (2019) highlighted that levels of burnout increase during the second semester. The time of data collection could explain why medical students had the highest level of burnout. Indeed, the first semester for first-year students is the beginning of students’ academic journey so their identification and relationships with others and teachers may not have had the time to fully develop. That is why further studies are needed to test levels of burnout from students in medicine and in other disciplinary at a single time point.

Third, this preliminary validation study does not provide cut-off scores. Indeed, several studies are needed to get cut-off score because the population must be the most representative of all students.

Finally, other studies may be necessary to adapt it to other contexts such as the work domain (e.g., Huyghebaert et al., 2018) and explore its criterion validity with other burnout measures validated and available in French such as the Burnout Assessment Tool (Schaufeli et al., 2020), the Burnout Measure Short version (Malach-Pines, 2005; Lourel et al., 2007) to cite a few. They are however less precise than our tool which assess in a same tool all sub dimensions. It would be also interesting to explore relations between our burnout measure and various antecedents and consequences that have already been identified in the literature in order to extend its nomological network.

### Practical implications

Despite these limitations, the creation and validation of the BIM for College Students could be useful to both practitioners and researchers. Indeed, it is not under copyright and thus easily accessible by all. To our knowledge, this is the first measure to simultaneously assess several targets of detachment and several types of weariness among college students. These distinctions could allow for a more precise representation of students’ burnout. Indeed, when focusing only on detachment toward studies as it is the case in the existing tools, information about relationships with other students and/or with teachers are missing. To only focus on studies does not allow getting information about their relation with other interpersonal targets (i.e., other students, teachers). However, this type of information is also important because other students and teachers are an important part of students’ environment.

The BIM for College Students could be an interesting tool for practitioners as they could adapt the prevention of burnout depending on the dimensions that students score the highest on. If students score high on all dimensions of burnout, practitioners could interview, counsel and assist them to avoid their dropping out of college. This latter point represents a huge implication for both students and the educational system. Finally, this tool could not only allow for the replication in the academic context for results found in the work context but also highlight specific causes or consequences specific of this academic context. Indeed, if we know of the numerous deleterious effects of school burnout such as cognitive determent, low academic performance, risky behaviors or dropout (Meier and Schmeck, 1985; Bask and Salmela-Aro, 2013; Walburg et al., 2014), we do not know their links with the different kinds of detachment. A better knowledge of those links might, again, allow a better prevention.

## Data availability statement

The raw data supporting the conclusions of this article will be made available by the authors, without undue reservation.

## Ethics statement

Ethical review and approval was not required for the study on human participants in accordance with the local legislation and institutional requirements. Written informed consent for participation was not required for this study in accordance with the national legislation and the institutional requirements.

## Author contributions

TW and SB contributed to studies conception and were in charge of data collection and statistical analyses. TW, SB, and TH-Z contributed to writing the first draft of the manuscript and approved the submitted version. All authors contributed to the article and approved the submitted version.

## Conflict of interest

The authors declare that the research was conducted in the absence of any commercial or financial relationships that could be construed as a potential conflict of interest.

## Publisher’s note

All claims expressed in this article are solely those of the authors and do not necessarily represent those of their affiliated organizations, or those of the publisher, the editors and the reviewers. Any product that may be evaluated in this article, or claim that may be made by its manufacturer, is not guaranteed or endorsed by the publisher.

## Footnotes

^1^The raw data supporting the conclusions of this article will be made available by the authors, without undue reservation.

^2^It was chosen not to allow covariances between errors because of scientific parsimony (Byrne, 2009), and because, according to Byrne, adding covariances between errors after having tested a model correspond to an exploratory rather than a confirmatory logic (Byrne, 2009, p. 89). Finally, the ‘burnout model 4’ already have good psychometric properties without allowing covariances between errors.

## Abbreviations

BIM: Burnout Integrative Measure
BMI: Burnout Model 1
BM2: Burnout Model 2
BM3: Burnout Model 3
BM4: Burnout Model 4
OS: Other students
TEA: Teachers
STU: Studies
COG: Cognitive
PHY: Physical
EMO: Emotional
INA: Inadequacy
DET OS: Detachment toward other students
DET TEA: Detachment toward teachers
DET STU: Detachment toward studies
COG WEA: Cognitive weariness
PHY WEA: Physical weariness
EMO WEA: Emotional weariness
